# Management of adrenocortical carcinoma in Slovenia: a real-life analysis of histopathologic markers, treatment patterns, prognostic factors, and survival

**DOI:** 10.2478/raon-2025-0013

**Published:** 2025-02-27

**Authors:** Urska Bokal, Jera Jeruc, Tomaz Kocjan, Metka Volavsek, Janja Jerebic, Matej Rakusa, Marina Mencinger

**Affiliations:** 1 Department of Medical Oncology, Institute of Oncology Ljubljana, Ljubljana, Slovenia; 2 Institute of Pathology, Faculty of Medicine, University of Ljubljana, Ljubljana, Slovenia; 3 Department of Endocrinology, Diabetes and Metabolic Diseases, University Medical Centre Ljubljana, Ljubljana, Slovenia; 4 Faculty of Medicine, University of Ljubljana, Ljubljana, Slovenia; 5 Department of Methodology, Faculty of Organizational Sciences, University of Maribor, Kranj, Slovenia

**Keywords:** adrenocortical carcinoma, Helsinki score, ENSAT stage, systemic treatment, survival, prognostic factors

## Abstract

**Background:**

Adrenocortical carcinoma (ACC) is a rare cancer that presents significant diagnostic and therapeutic challenges. We analyzed the management and estimated survival of ACC patients in Slovenia over a 17-year period.

**Patients and methods:**

Patients registered in the National Cancer Registry and treated from 2000 to 2017 were included. The survival and prognostic factors were assessed using the Kaplan-Meier method and Cox regression, respectively.

**Results:**

Forty-eight patients were included in our analysis. At the time of diagnosis, 6%, 42%, 25% and 27% had stage according European Network for the Study of Adrenal Tumors (ENSAT) I, II, III and IV, respectively. Adjuvant treatment with mitotane was assigned to 18 of 34 potentially eligible patients. High-risk patients treated with adjuvant mitotane showed a reduced probability of death, although the difference was not statistically significant. Relapses had numerically higher rate of R1 resection and higher Ki67. Eleven patients underwent first-line therapy with etoposide, doxorubicin, cisplatin and mitotane (EDP-M). Their median progression-free survival was 4.4 months. The median overall survival of entire cohort was 28.9 and the median disease-specific survival (DSS) was 36.2 months. The 5-year DSS rate of ENSAT I, II, III and IV were 100%, 56%, 50% and 0%, respectively. The prognostic value of ENSAT stage and Helsinki score regarding overall survival was confirmed with the multivariate analysis.

**Conclusions:**

The 5-year DSS of our ENSAT II patients was worse than reported in contemporary cohorts. Suboptimal surgery and inconsistent adjuvant therapy with mitotane might have contributed to this outcome. Better outcomes of this rare disease might be accomplished with dedicated teams including various specialties, working towards optimal staging, diagnostic and therapeutic measures.

## Introduction

Adrenocortical carcinoma (ACC) is an aggressive orphan tumour. The annual incidence is around two cases per million people.^1^ The postoperative disease-free survival rate at five years is less than 50% and the 5-year survival rate for metastatic disease worldwide remains dismal.^2^ About 50–60% of patients with ACC have clinical hormone excess. In most cases, hypercortisolism (Cushing′s syndrome) and/or virilisation syndrome due to androgen secretion are observed.^3^

As clinical, laboratory, and imaging features of ACC overlap with other benign and primary or secondary malignant adrenal tumours, the final diagnosis and malignant potential of an adrenal lesion depends largely on sophisticated histopathologic analysis of the surgical specimen. To facilitate and standardize the diagnosis of ACC, several multiparametric scoring systems have been developed based on combined histopathological features, such as the Weiss score and the Helsinki score.^4^ The Weiss score considers nine histopathologic parameters and remains one of the most used scoring systems in clinical practice to classify conventional ACC in adults.^5^ A more recently developed score, the Helsinki score, focuses on a combination of the Ki67 proliferation index, mitotic rate, and the presence of necrosis. It can be used not only for the diagnosis of conventional ACC, but also for oncocytic and myxoid variants.^6^ Two staging systems have been also proposed: TNM staging, which was revised in 2017 (AJCC cancer staging^7^), and the staging system by the European Network for the Study of Adrenal Tumors (ENSAT) in 2009.^8^

According to current clinical practice guidelines, all patients with ACC and a high risk of recurrence after surgery (ENSAT stage III, R1 resection or Ki67 >10%) should receive adjuvant treatment with mitotane.^3^ Recently published results of the ADIUVO trial did not support adjuvant treatment with mitotane in patients with low-intermediate risk of recurrence (ENSAT stage I-III, R0 resection and Ki67 ≤ 10%).^9^ Only one phase III clinical trial (FIRM-ACT) was conducted in patients with ACC. In this trial, etoposide, doxorubicin, and cisplatin (EDP) plus mitotane resulted in higher response rates and longer progression-free survival than streptozocin plus mitotane as first-line therapy, although there was no significant difference in overall survival.^10^ Recently, immune check point inhibitors and cabozantinib have been used successfully in some patients with ACC.^11,12^ Other treatment options are experimental at best.^13^

Locoregional therapies are recommended for slowly progressive oligometastatic disease or when a sustained response to systemic therapy has been achieved. In addition to surgery, there are other options such as radiotherapy, radiofrequency ablation and chemoembolization.^3,14^

We performed a retrospective analysis including histopathologic assessment of primary tumours and metastases, systemic treatment patterns, and outcomes of our patients with ACC over nearly two decades (2000-2017). In addition, we identified factors influencing survival.

## Patients and methods

**Figure 1.**
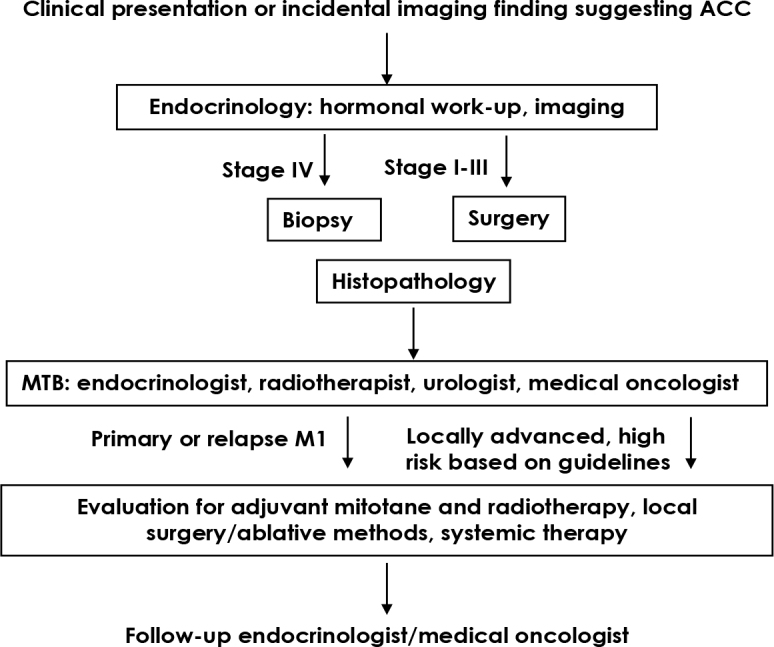
A patient flowchart describing the process of diagnostics and treatment decision-making. ACC = adrenocortical carcinoma; MTB = multidisciplinary tumour board; M1 = metastatic disease

We conducted a retrospective cross-sectional study including all adult patients who were diagnosed with ACC from year 2000 to 2017 and were treated at the University Medical Centre Ljubljana and the Institute of Oncology Ljubljana. The patient list was taken from the National Cancer Registry. The study protocol was approved by the Review Board and Committee for the Medical Ethics of the Institute of Oncology in Ljubljana (ERIDEK - 0024/2020). A flowchart detailing the diagnostics and treatment decision-making process is shown in Figure 1.

We used patients′ medical records to collect their demographic and clinical parameters, data on ENSAT stage at diagnosis, tumour size (defined as the largest diameter in axial plane), biochemically confirmed hormone hypersecretion, surgical treat-ment of the primary tumour, status of resection margins, adjuvant radiotherapy and adjuvant mitotane treatment. We explored options for the first, the second and the third line of systemic treatment. Survival analyses and tests for prognostic factors were performed for the entire cohort. In accordance with our sample size, we chose to determine the prognostic significance of three variables: ENSAT stage (I and II versus III and IV), Helsinki score and hypercortisolism (present versus absent). The prognostic value of adjuvant mitotane treatment was analysed for the high-risk patients.^3^ In addition, the prognostic value of the Ki67 index in metachronous metastasis or local recurrence (20 or more versus less than 20) was tested in univariate analysis. The cut-off value of 20 was used in analogy to the cut-off values for primary tumours.^15,16^

Two additional assessments were carried out. First, data on local treatment options for metastatic/locally recurrent disease were analysed in detail. Several survival parameters were calculated in patients who underwent surgery for local recurrence/oligometastatic disease. The median treatment-free interval (mTFI) was defined as the time from diagnosis of ACC to resection of the first metachronous metastasis. The median progression-free survival (mPFS) was defined as the time from the local therapy of the first metastasis to the systemic disease progression or death. The median overall survival of this cohort (mOS) was defined as the time from diagnosis of ACC to death.

Second, two experienced pathologists (MV and JJ) reviewed all available archival histologic tissue samples of primary tumours and metastases of the included patients. If multiple metastases or local recurrences were available, only the one that had been histologically analysed first was revised. The Weiss scoring system was used in all adrenalectomy specimens except for the two patients with the oncocytic variant of ACC in which Weiss system tends to overdiagnose malignancy and Lin-Weiss-Bisceglia criteria are applied instead.^17^ The Weiss system was only used to evaluate adrenalectomy specimens, as it is not possible to perform the required assessment of venous or capsular invasion in other types of specimens (e.g. needle biopsy, metastasis, excision of recurrent disease).

The Helsinki index and the Ki67 proliferation index were determined using visual estimation method.^18^ Immunohistochemistry for Ki67 antigen (clone MIB-1, Dako, 1/50, UltraView) was performed using a Benchmark XT Ventana system according to manufacturer′s instructions. The correlation between the Weiss and the Helsinki score of the primary tumours was investigated. The Ki67 index and the Helsinki score of both specimens were compared in patients in whom the tissue from both, the primary tumour and the first local recurrence or metastasis was available.

### Statistical analyses

Data for continuous variables were presented as median and range and for categorical data as frequencies and percentages. The cut-off date for the survival analysis was October 15, 2020. Survival outcomes were calculated using Kaplan-Meier method with 95% confidence intervals and predictors of survival were calculated using Cox proportional hazards regression models. The p-values shown are two sided and the p-value < 0.05 was considered statistically significant. The calculations were performed using the statistic software package IBM SPSS 28.0. Correlation between Weiss and Helsinki score of primary tumours was investigated with Spearman′s rank correlation coefficient test.

To further explore the prognostic power of Helsinki score receiver operating characteristic (ROC) analysis was done. Area under the ROC curve (AUC) was calculated to assess the ability of Helsinki score to differentiate between “alive” versus “death” status. The cut-off value of Helsinki score to differentiate between different prognostic groups was determined based on maximizing the Youden index in the context of the ROC curve.

## Results

### Patients’ characteristics

Forty-nine adult patients were diagnosed with ACC at our two centres during the studied period (2000–2017). During histologic revision one patient was diagnosed with adenoma instead of ACC and was excluded from the analysis. Characteristics of all analysed patients and of the 20 patients who relapsed after radical surgery are shown in Table 1. One patient with ENSAT stage III was not treated with radical surgery as the tumour was considered inoperable.

**TABLE 1.** Characteristics of all analysed patients and of the patients with European Network for the Study of Adrenal Tumors (ENSAT) I-III that relapsed after surgery with curative intent

Characteristics		All included N = 48 (%)	Relapsed N = 20 (%)
Age: median (range); years		56.6 (21–82)	54.0 (21–72)
Sex	Male	21 (44)	11 (55)
	Female	27 (56)	9 (45)
ENSAT stage at diagnosis	I	3 (6)	0
	II	20 (42)	12 (60)
	III	12 (25)	8 (40)
	IV	13 (27)	N/R
Tumour size: median (range), cm	12 (4–30)		12.5 (5–30)
	Unknown	6	2
Hormone secretion	Yes – GC*	17 (35)	5 (25)
	Yes – O	8 (17)	4 (20)
	No	20 (42)	11 (55)
	Unknown	3 (6)	/
Weiss score (median, range)	6 (4–9)		7 (5–9)
	N/D^+^	15	1
Ki67 score** (median, range)	20 (1–70)		24 (8–60)
	N/D°	9	1
Helsinki score** (median, range)	28 (1–78)		31 (16–68)
	N/D°	9	1
Resection margins of patients	RO	26 (76)	17 (85)
stage I –III treated with curative	R1	5 (15)	3 (15)
surgery	Rx	3 (9)	/

1 GC = glucocorticoids; O = other; N/D = not determined; N/R = not relevant;

1 * isolated or in combination with other hormones;

1 ** of primary tumour;

+ due to oncocytic variant (2), unavailability of tissue samples (3): primary not operated - 1 patient; tissue not available at our institutions - 2 patients), only fine needle (6) or core needle biopsy (4) of primary tumour or metastases;

° due to unavailability of tissue samples (3) or only fine needle biopsy of primary tumour or metastases (6)

**Figure 2.**
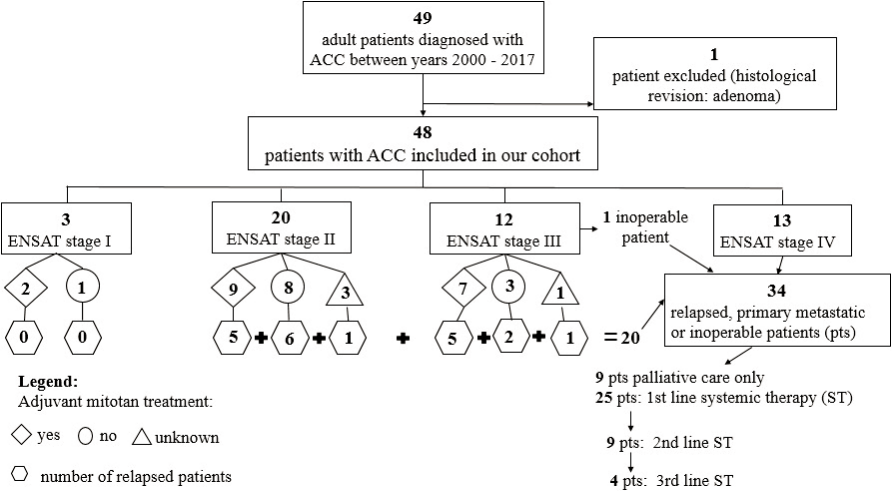
Our cohort according to European Network for the Study of Adrenal Tumors (ENSAT) stage, adjuvant mitotane treatment, relapses and different lines of systemic treatment. ACC = adrenocortical carcinoma; ST = systemic treatment

Figure 2 shows our cohort according to ENSAT stage, adjuvant mitotane treatment, relapses and different lines of systemic treatment.

### Adjuvant mitotane treatment

Thirty-four patients, who were classified as ENSAT stage I-III underwent surgical removal of the primary tumour and could have been considered eligible for adjuvant mitotane. However, 12 patients (35.3%, median age 49.5 years; 7 females), including 6 patients at high risk of recurrence after surgery (Ki67 >10%^3^); of whom three were classified as ENSAT stage II and three as stage III, were not started on this treatment. Relevant data was absent for further 4 patients (11.8%). The remaining 18 patients (52.9%, median age 63 years; 9 females,) received mitotane. Half of the treated subgroup (or 9 patients) were classified as ENSAT stage II, 38.9% (or 7) as stage III, and 11.1% (or 2) as stage I. Ki67 was > 10% in 14 of these patients (77.8%) with missing data for one patient in ENSAT stage III. Collectively, only two patients at low/intermediate risk of recurrence after surgery (R0, Ki67 ≤ 10%^3^; classified as ENSAT stage I and stage II), received mitotane.

Two patients were followed at another institution, so further data about mitotane treatment was available for 16 patients. Median time from surgery of primary tumour to start of adjuvant mitotane was 26.5 (range 6–126) days, while median duration of treatment was 17.4 (3–73) months with the median daily mitotane dose of 2750 (500 – 7000) mg. All patients were on concurrent hydrocortisone replacement therapy with median daily dose of 40 (15–45) mg. Three patients (18.7%) progressed when on mitotane after median time of 30 (10–31) months of treatment and one patient died of metastatic breast cancer in the 11th month of adjuvant mitotane treatment. All discontinuations of mitotane during adjuvant treatment were permanent. Reason for stopping were adverse effects: gastrointestinal in 2 (25.0%), hepatic in 2 (25.0%) neurocognitive in 1 (12.5%) and other in 3 (37.5%) cases. Data on mitotane plasma concentrations were not obtainable for most patients, so they were not included in the analysis. High-risk patients that received adjuvant mitotane had lower risk of death (HR 0.614, 95% CI 0.207-1.820), but the difference was not statistically significant (p = 0.379).

### Adjuvant radiotherapy

Thirty-four patients classified as ENSAT stage I-III underwent surgical removal of the primary tumour. Of these 34, 16 had either R1 resection or/and ENSAT stage III disease. One of these 16 patients had both ENSAT III and R1 resection, 11 had ENSAT III and 4 had R1. Finally, only three of them received adjuvant radiotherapy (RT): one with R1 resection, one with ENSAT III and one with both criteria. All other potential candidates from this group did not receive adjuvant RT (one of them due to treatment refusal); but two other patients without criteria did.

### Systemic treatment regimens for inoperable locally advanced or metastatic disease

Thirty-four out of 48 patients had inoperable locally advanced or metastatic disease, either at the time of the primary diagnosis or recurring after surgery. Nine patients were referred to palliative care only. First-line systemic treatment regimens for the remaining 25 patients are listed in Table 2.

**TABLE 2.** First-line systemic treatment regimens for inoperable locally advanced or metastatic disease

Treatment regimen	Patients (N)
EDP-mitotane	11
mitotane (+/- local therapy)	11
etoposide + carboplatin	1
dacarbazine + cyclophosphamide + vincristine	1
tamoxifen	1

1 EDP = etoposide, doxorubicin and cisplatin

The median age of 11 patients who were treated with standard first-line chemotherapy (EDP-mitotane) was 56 years (range 29–70). Their performance status was 0 in 6 patients (54%), 1 in 4 patients (36%) and 2 in 1 patient (9%). Median number of cycles received was 5 (range 2–7). The mPFS was 4.4 months (95% CI 1.5–7.3) and the mOS was 15.8 months (95% CI 7.7–23.8). Two patients achieved partial response (PR), 6 patients had stable disease (SD), and 3 patients had progressive disease (PD). There were no complete responses.

The patient who received treatment with dacarbazine, cyclophosphamide and vincristine were initially diagnosed with pheochromocytoma, but histologic revision from a highly specialized centre confirmed the diagnosis of ACC.

All 25 patients treated with first-line therapy progressed during the therapy or follow-up period; among them, 9 (36%) received 2nd line systemic treatment, which is listed in Table 3 together with responses achieved.

**TABLE 3.** Second line treatment regimens

Treatment	Patients (N)	Response
gemcitabine + capecitabine +/- mitotane	5	SD: 1 PD: 4
EDP-mitotane	1	PR
pembrolizumab	1	SD
dacarbazine + capecitabine + imatinib	1	PD
vinblastine + interferon alpha-2a	1	PD

1 EDP = etoposide, doxorubicin and cisplatin; SD = stable disease, PD = progressive disease, PR = partial response

Five patients who received second line therapy with gemcitabine and capecitabine had median number of 2.5 cycles (range 2-5), median progression free survival 2.3 months (95% CI 1.5-3.1) and median overall survival 10.0 months (95% CI 1.9– 18.1).

Third line therapy was prescribed to 4 patients: reintroduction of gemcitabine - capecitabine, metronomic therapy with cyclophosphamide, thalidomide plus mitotane, all of whom progressed. One patient received radionuclide therapy with ^131^I-iodometomidate in a highly specialised centre in Würzburg, Germany, and had survived for 8 months after referral.

### Survival rate of ENSAT stage I, II, III and IV

The 5-year overall survival (OS) of patients with ENSAT stage I, II, III and IV was 100%, 50%, 50% and 0%. If stages I/II and III/IV were grouped together the 5-year OS was 56.5% and 24%. The 5-year disease specific survival (DSS) was 100%,56%, 50% and 0%, respectively. The 5-year OS of patients with ENSAT stage I-III who were diagnosed before year 2010 was 61.9% and of patients with ENSAT stage I-III who were diagnosed after the year 2010 was 42.9%; the difference was not statistically significant (p = 0.132). The mOS of patients with ENSAT stage IV who were diagnosed before and after year 2010 was 1.5 months (95% CI 0.00 – 3.89) and 8.6 months (95% CI 0.42 – 16.73), respectively. This difference was also not statistically significant (p = 0.338).

### Survival analysis of the whole cohort and prognostic factors

**Figure 3.**
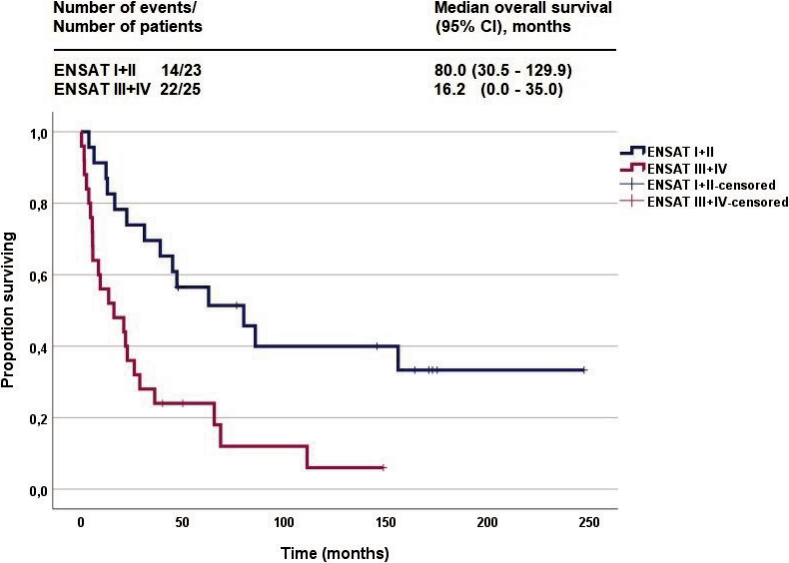
Kaplan-Meier curves of overall survival according to European Network for the Study of Adrenal Tumors (ENSAT) stage.

The median follow-up of the cohort was 30.0 months; 36 (75%) patients died. The mOS was 28.9 months (95% CI 10.25-47.51). Three patients died for other reasons (not ACC). Median DSS was 36.2 months (95% CI 11.8-60.6). In univariate analysis significant impact of ENSAT stage III/IV versus I/II (HR 2.989; 95% CI 1.483-6.023; p = 0.002) and Helsinki score (HR for each additional unit of Helsinki score 1.02; 95% CI 1.003–1.042; p = 0.021) on OS was confirmed, but not of hypercortisolism (HR 1.523; 95% CI 0.772–3.006; p = 0.225). Multivariate analysis confirmed the prognostic value of the ENSAT stage (HR 2.796; CI 95% 1.258– 6.212; p = 0.012) and Helsinki score (HR 1.027; 95% CI: 1.005–1.049; p = 0.015). Kaplan-Meier curves of OS according to ENSAT stage groups are shown in Figure 3.

Fourteen of 34 patients who were operated on ACC ENSAT stage I-III remained disease free. Two patients progressed more than 5 years after surgery on primary tumour: 10 years and 9 months with local recurrence treated with surgery and RT, being alive at the time of the data cut-off; 5 years and 7 months with inoperable local recurrence, later further systemic progression and death.

To further explore the prognostic power of Helsinki score ROC analysis was performed which was statistically significant (overall model quality 0.55) with AUC 0.761 (95% CI 0.551-0.971). The cutoff value for Helsinki score determining two prognostically different groups was 19.5.

### Locoregional treatment for primary metastatic or relapsing disease

Only 6 patients from our cohort were treated for local recurrence or metastatic disease with local treatment methods. As a rule, surgery was performed; which was combined with radiofrequency ablation (RFA) in only one patient. Patients who received only palliative radiotherapy were not included in this subgroup analysis. Most patients (5 out of 6 or 83%) had metachronous metastases that had occurred three months or more after surgery for the primary tumour.^19^ One patient had solitary synchronous liver metastasis that was resected concomitantly with the primary tumour. In another patient, surgery was performed multiple times and combined in the third session with RFA of two liver metastases and one thoracic metastasis that had spread through the diaphragm.

Overall, local recurrences were resected in three (50%) patients, liver metastases in two (33%) patients and lung and vertebral metastases in one (16%) patient. Four patients: two with local recurrence, one with liver metastasis and one after right pneumonectomy of multiple lung metastases received RT after the local surgery. The mTFI was 32.1 months (95% CI 17.6–131.3). The mPFS was 7.29 months (95% CI 0.00-61.2) and mOS was 65.5 months (95% CI 9.4-121.55). The two patients who received RT after surgery for local recurrence both remained disease-free.

### Histopathologic features

Histopathologic analysis was possible in 40 of our patients where the diagnosis was confirmed histologically by either resection or core needle biopsy of the primary tumour or metastases. In six patients, the primary tumour or metastases were only verified cytologically and in two patients adrenalectomy was performed but no tissue was available for analysis at our two institutions. Archival tissue blocks of formalin-fixed paraffin-embedded (FFPE) tissue for analysis were obtained after adrenalectomy (35: from 34 patients ENSAT stage I–III and one patient ENSAT stage IV), core needle biopsy of primary tumours (4) or metastatic deposits (2) and resection of the first local recurrences or metastases (10).

Histologic variants of ACC in our cohort were as follows: 35 had conventional ACC, two patients had oncocytic variant, two myxoid (one of them partial myxoid and partial conventional type), and one sarcomatoid variant. Survival of patients with pure myxoid (3.9 months) and sarcomatoid (6.5 months) histologic variant was less than the medium overall survival of the whole cohort (28.9 months). Both patients with oncocytic variant and the patient with partial myxoid variant (ENSAT stage I) were progression-free at the time of the data cut-off.

As far as primary tumours were concerned, Weiss score was determined in 33 patients and Ki67 proliferation index and Helsinki score in samples from 39 patients. These data are shown in Table 1. In 12 metastatic/recurrent samples, the median Ki67 index was 27.5 (range 11–60) and the median Helsinki score was 35.5 (range 19–68). There was no correlation between Weiss and Helsinki scores of primary tumours as the Spearman′s correlation coefficient was - 0.092 (p = 0.612). In 11 patients, the tissue from resected primary tumours as well as from the first metastasis/local recurrence was available for investigation. Table 4 shows the comparison between their Ki67 index and Helsinki score.

**TABLE 4.** Ki67 proliferation index and Helsinki score shown for primary tumour (P) and first metastasis/local recurrence (M)

Patient	Ki67		Helsinki score	
	P	M	P	M
1	16	40	24	48
2	20	16	23	24
3	15	25	23	33
4	20	20	28	23
5	30	30	38	38
6	40	30	48	38
7	15	11	18	19
8	30	20	38	28
9	10	11	18	19
10	40	50	48	50
11	25	50	29	58

Patients from 1 to 9 had local recurrence or metachronous metastasis. In the univariate analysis of this cohort those with Ki67 index of the local recurrence/metastasis of 20 or more (N = 6) had a statistically significantly shorter survival from the diagnosis of being metastatic/recurrent than the others (N = 3), HR 1.12 (95% CI 1.01 – 1.25), p = 0.033. Multivariate analysis was not performed due to small sample size.

## Discussion

We have confirmed the poor prognosis of patients with ACC treated in routine clinical practice. In almost half of our patients, the tumour was confined to the adrenal gland and less than one third had primary metastatic disease, which differs from the stage distribution in historic reports. In an older series of 42 patients diagnosed with ACC at Roswell Park Memorial Institute between 1929 and 1977, only 7% of patients had tumour confined to the adrenal gland, while 41% had locally advanced disease and 52% had metastatic disease.^20^ Wooten *et al*. reviewed data on ACC patients described in the English literature between 1952 and 1992 and found that only 31.8% of 608 patients had tumours confined to the adrenal gland.^21^ However, in contemporary reports from Portugal and Finland, stages were distributed similarly to our cohort, with 43% and 59% of tumours confined to the adrenal gland, respectively.^22,23^ The observed contemporary shift in ENSAT staging is likely due to earlier ACC diagnosis, resulting from better availability of radiologic imaging, often performed for unrelated reasons (adrenal incidentalomas).^23^

In our series oncocytic and myxoid variants accounted for five percent of ACCs, while sarcomatoid variant was detected in 2.5% of all ACC included in the histopathologic analysis. The relative frequency of the variant histology is consistent with previously published data.^4^ As expected, the clinical behaviour of patients with myxoid and sarcomatoid variants of ACC was worse than in patients with the classic variant and the behaviour of oncocytic ACC was better. The patient with the partial myxoid variant who was progression-free at the time of the data cutoff, was diagnosed as ENSAT stage I, which was probably the most important factor for their good prognosis. Presumably, favourable stage distribution and access to systemic treatments impacted the median OS of our entire cohort (28.9 months), which is longer than observed historically (14 months).^20^ Five-year overall survival rate of our ENSAT stage III (50%) and IV (0%) patients is comparable to published series from Portugal^22^ (56%; 0%) and Finnland^23^ (stage III/IV 24% for our cohort *vs*. 26%). On the other hand, five-year survival rate of our ENSAT stage II patients is inferior to both Portuguese (stage I/II 56.5% *vs*. 67%) and especially to Finnish cohort (stage I/II 96%). Worse outcome can be at least partially explained by incomplete resections^24^ (four patients with ENSAT stage II had R1 resection), less than optimal surgical technique by non-expert surgeon, e.g. not performing concomitant regional lymphadenectomy (four out of five patients operated by non-urologists relapsed), and lack of adjuvant mitotane therapy (three patients with stage II should receive it due to high Ki67 but did not). In addition, some of our early ENSAT stage II patients might have been misclassified due to suboptimal staging, e.g. performing a chest X-ray instead of a CT. A higher percentage of stage I tumours in the Finnish cohort (19% versus 6% in our cohort) might have been also partially responsible for the difference. The five-year overall survival of our ENSAT I-III patients diagnosed after 2010 was not better than before 2010. Less favourable stage distribution without any ENSAT stage I patients diagnosed after year 2010 might have contributed to the lack of improvement. This indicates that during our observed period 2000 – 2017 there was no trend in detecting disease earlier; this trend was only observed in comparison to historic cohorts as discussed previously.

In the Finnish cohort, 79% of patients received adjuvant mitotane therapy, which was reported as a factor associated with better survival in this study.^23^ Mitotane was prescribed to everybody after successful surgery except in cases with a very low risk for recurrence according to an expert opinion.^25^ No such straight-forward reasoning was present in our cohort. Only 52.9% of the patients started therapy with mitotane, therefore, 6 patients at high risk of recurrence after surgery^3^ might have been inappropriately excluded from this treatment. The main reason for this undertreatment was a lack of clinical practice guidelines on the management of ACC^3^ during the observed period causing not only uncertainties in the mitotane use, but also patients’ refusal of this treatment in some cases. Interestingly, similar inconsistency was also apparent in a recent Italian national cohort study where among 134 operated ACC patients selected just for surveillance 44.4% had Ki67 > 10%.^26^ On the positive side, only two of our patients who were started on mitotane were at low/intermediate risk of recurrence after surgery and might have been overtreated.^9^ Furthermore, most of our patients started with mitotane within the ideal 6 weeks after surgery. The drug was mostly administered for at least two years, but no longer than 5 years, as recommended.^3^ Some patients did not follow this pattern and there were 9 permanent discontinuations due to adverse effects like in other cohorts.^22,23^ Hydrocortisone supplementation was a uniform feature of all our patients on mitotane. However, our daily hydrocortisone replacement doses (median 40 mg, range 15–45 mg) might not have been entirely sufficient, as these patients typically require 50 mg or even up to 100 mg daily due to increased hydrocortisone clearance and increased cortisol-binding globulin.^25,27^ If our highrisk patients received adjuvant mitotane, they had better survival, as it was previously shown else-where.^23^ A lack of statistical significance could be attributed to small size of our cohort.

The most frequent combined chemotherapy used for the first line treatment was EDP-M protocol, which is the suggested treatment by the guidelines^3,14^ according to the results of FIRM-ACT trial.^10^ The outcomes of our patients who received the first line EDP-M treatment in real-life clinical practice were comparable to the results of that trial (mPFS 4.4 months (95% CI 1.5–7.3) versus 5.0 months (95% CI 3.5–6.9), mOS 15.8 months (95% CI 7.7-23.8) versus 14.8 months (95% CI 11.3–17.1), which probably reflects the fact that only patients with a good performance status were treated in such way (mostly WHO PS 0/1 and only 1 patient WHO PS 2). Monotherapy with mitotane was used as frequently for the first line therapy as the EDP-M protocol. Among reasons for the monotherapy with mitotane were poor performance status, comorbidities and patients’ refusal of chemotherapy. No comparison between EDP-M protocol and mitotane monotherapy could be made since patients in the latter group were in worse general health.

Further lines of treatment were poorly effective, and few patients were able to receive them (36% of patients treated with the first line and 44% of patients treated with the second line therapy). In this setting, there is no proven systemic therapy showing improved survival in a randomised controlled trial. Accordingly, the selection of second-line treatment for our patients was based on small phase 2 trials or even case reports.^28,29^

The mOS of patients who received systemic treatment for advanced disease was 13.0 months (95% CI 5.1–20.8) which is less than the mOS of 18.7 months that was observed by the Ohio State University Comprehensive Cancer Centre between years 1997 and 2016.^30^ In their cohort 64% of patients received the second line treatment and they also had the possibility to participate in clinical trials. This emphasizes the importance of collaboration with international specialised centres when treating this rare disease.^31^ In our cohort the mOS of patients with ENSAT stage IV diagnosed after year 2010 is higher in comparison to those who were diagnosed until year 2010 which may reflect better systemic treatment options in the recent decade, although the difference is not statistically significant. Two patients experienced relapse of the disease more than 5 years from surgery of the primary tumour, which supports the continuation of follow-up beyond 5 years as suggested by the clinical guidelines.^3^

Only six patients were treated with local therapy for relapsing/metastatic disease. Five patients had surgical resection of their solitary metastatic lesions according to the guidelines where routine use of surgery in widespread disease is not recommended.^3^ The remaining patient who underwent surgery despite several synchronous metastases died after only three months reflecting the futility of the approach due to more aggressive disease. Contrary to that, the other 5 patients had a slowly progressive disease as indicated by mPFS that was 7.3 months and mTFI that was 31.1 months.

Two patients had been disease-free for more than 10 years after surgery of local recurrence, which further dictates a tight follow up with an early detection of resectable local recurrence to benefit some patients. Both patients with long lasting remission received also postoperative radiation. A large recent study in advanced ACC provided evidence that RT can be effective.^32^

Importantly, only 3 out of our 5 patients who received adjuvant RT, were appropriately selected according to current guidelines. On the other hand, 9 of 13 patients classified as ENSAT stage III and/or having R1 resection were not offered adjuvant RT after surgery for a primary tumour. According to current guidelines adjuvant RT should be considered on an individual basis (in addition to mitotane) in patients with R1 or RX resection or/and in ENSAT stage III.^3^ Retrospective data showed that adjuvant RT can reduce the risk of local recurrence but does not prevent distant recurrences and, consequently, does not impact OS.^32^ Randomised data on the usefulness of RT after surgical resection of primary tumour and of metastases are needed.

Only a single patient underwent RFA. Other locoregional methods such as stereotactic radiation or chemoembolization of metastases were not used. Contrary to our approach, it is currently recommended to use several local therapeutic measures on an individual basis in addition to surgery for advanced ACC.^3^ Furthermore, a recent retrospective analysis of 106 patients supported the use of locoregional treatments to treat ACC recurrence.^33^ It is reasonable to assume that close collaboration with an interventional radiologist could have optimised palliation in a larger proportion of our patients with metastases amenable for local treatment.

Not only the disease stage, but also marginfree (R0) resection, glucocorticoid excess and Ki67 proliferation marker were suggested as prognostic factors of survival.^14^ Due to small sample size only ENSAT stage, Helsinki score and hypercortisolism were tested as prognostic factors. In multivariant analysis, both ENSAT stage and Helsinki score predicted survival. Helsinki score was validated as a prognostic marker for ACC in several other studies.^6,34,35^ Unlike ENSAT stage and Ki67, Helsinki score was not found to have prognostic value in a recent series of patients with ACC from Finland.^23^ Helsinki score includes two proliferation markers (Ki67 immunohistochemistry and mitotic count) and necrosis. While the prognostic value of proliferation has been validated in many studies on the Weiss parameter^15,36,37,38,39^, the presence of necrosis, on the other hand, has only recently been suggested as the most powerful ominous factor and the best predictor of OS and DFS in ACC patients.^40^

No correlation between Helsinki and Weiss score was found in our cohort, which is different from the findings of another study showing strong positive correlation between these two scoring systems.^41^ In addition to low number of patients included in our calculation, technical issues with respect to Ki67 immunohistochemistry on archival samples not allowing optimal evaluation could partially explain these discrepancies.

In our cohort, the cut-off value for the Helsinki score of 19.5 performed best in terms of prognostic stratification. In comparison, Pennanen *et al*. proposed a lower cut-off value of 17 to distinguish tumours with prolonged survival from rapidly progressing tumours.^34^ Duregon *et al*. used the Helsinki scores of 13 and 19 to classify patients into three prognostically distinct groups.^6^ In a more recent study, the Helsinki score of 20 was identified as one of the strongest independent predictors of death, being able to distinguish tumours with prolonged survival from those with rapid progression.^35^ In our cohort, there were not enough patients with low Helsinki scores to allow stratification into three groups. Several patients with a high Helsinki score had a favourable clinical course, possibly due to relatively small tumour size, complete surgical resection, and good response to treatment. Based on our and similar studies, there is probably no exact cut-off value for the Helsinki score to prognosticate disease, but rather a range between 17 and 20.

We did not find a general tendency towards a higher Ki67 index in metastases compared to primary tumours, as it was shown by study investigating the Ki67 index in primary breast cancer and corresponding metastases.^42^ Nevertheless, previously published data for primary ACC tumours^15,16^ and our analysis of the Ki67 index of the first local recurrence/metachronous metastasis showed that a Ki67 index < 20 might correlate with a slower progression compared to Ki67 ≥ 20. There is similarity to what was demonstrated in breast cancer, where a low Ki67 index in metastasis was associated with longer survival independently of primary tumour proliferation.^43^ Beside TFI indicating aggressiveness of a disease course, analysis of Ki67 in a metastasis may be beneficial to indicate slowly progressing disease as has already been suggested in breast cancer.^43^ However, more data are needed to draw any firm conclusions.

Our study has some limitations due to its retrospective methodology and incomplete information from patient charts. In addition, archived FFPE material of varying quality and age, originating from different institutions had to be re-examined. Importantly, patients treated with mitotane were not compared according to their mitotane plasma concentrations due to missing data. Small sample size allowed only few prognostic factors to be tested.

The main strength of our study is the joint effort of pathologists, endocrinologists and medical oncologists to comprehensively review the management of ACC in Slovenia over the last two decades. We tried to highlight the available good practices while also exposing the shortcomings. In particular, the importance of the appropriate histopathology diagnosis and strict adherence to the clinical guidelines if available were pointed out to improve all aspects of management from expert surgery and adjuvant mitotane treatment to locoregional therapies.

## Conclusions

Research on ACC is partially hampered by the rarity of this type of cancer. Therefore, the presented real-world data might help the clinicians to improve the management of this rare and often fatal disease. A multidisciplinary approach, as highlighted here, is of paramount importance, and has already been shown to impact survival.^44^
